# Sporting activity after craniosynostosis surgery in children: a source of parental anxiety

**DOI:** 10.1007/s00381-020-04723-2

**Published:** 2020-06-11

**Authors:** Oloruntobi Rotimi, Gu-Yun Paul Jung, Juling Ong, N. U. Owase Jeelani, David J. Dunaway, Greg James

**Affiliations:** 1 Department of Surgery, Medway Maritime Hospital, Kent, UK; 2 UCL Medical School, University College London, London, UK; 3 Craniofacial Unit, Great Ormond Street Hospital, London, UK; 4 Great Ormond Street Institute of Child Health, University College London, 30 Guildford Street, London, WC1N 1EH UK

**Keywords:** Craniosynostosis, Craniofacial surgery, Sports, Anxiety, Quality of life

## Abstract

**Purpose:**

Craniosynostosis correction involves major skull surgery in infancy—a potential source of worry for parents when their treated children begin involvement in sports.

**Methods:**

Electronic multiple choice survey of parents of children who had undergone craniosynostosis surgery in infancy using 5-point Likert scales.

**Results:**

Fifty-nine completed surveys were obtained from parents of children who had undergone previous craniosynostosis surgery. Mean age of children was 7.8 years (range 3 months to 22 years), with 36 non-syndromic and 23 syndromic cases. The most common surgery was fronto-orbital remodelling (18). Fifty-two of 59 were involved in athletic activity. The most intense sport type was non-contact in 23, light contact in 20, heavy contact in 4 and combat in 5. Participation level was school mandatory in 12, school club in 17, non-school sport club in 21 and regional representative in 2. One child had been advised to avoid sport by an external physician. Mean anxiety (1–5 Likert) increased with sport intensity: non-contact 1.7, light contact 2.2, heavy contact 3.5 and combat 3.6. Twenty-nine of 59 parents had been given specific advice by the Craniofacial Team regarding athletic activity, 28 of which found useful. Three sport-related head injuries were reported, none of which required hospitalisation.

**Conclusion:**

Little information exists regarding sports for children after craniosynostosis surgery. This study suggests that parental anxiety remains high, particularly for high impact/combat sports, and that parents would like more information from clinicians about the safety of post-operative sporting activities.

## Introduction

Craniosynostosis is defined as the early closure of one or more cranial sutures and can cause head shape abnormalities with both functional and appearance-related consequences [1, 2]. Although exact timings and techniques vary between centres, many children referred to specialist centres undergo surgical correction in early childhood.

Taking part in play, sport and exercise is important for children’s development, enjoyment and health [3]. However, we noted anecdotally that many parents attending surgical follow-up expressed concern regarding children who had undergone surgical craniosynostosis correction in infancy to take part in sports, due to concerns about injury. In the population at large, there is increasing concern about impact head injury in sports [4–6]. In addition, there is increasing realization that parents have particular anxieties when a child is diagnosed with craniosynostosis [7, 8]. One study reported no traumatic brain injury in the 2 years following craniosynostosis correction [9]. However, a PubMed search did not yield any literature on the specific question of sporting or athletic activity following craniosynostosis surgery. We therefore decided to survey parents of children with craniosynostosis regarding anxiety in undertaking sporting activity.

## Methods

Participants were selected at random from operative electronic database and outpatient craniofacial clinics, from those who had undergone at least 1 craniosynostosis surgery. Parents were asked to complete a confidential electronic questionnaire (Google Forms). Data was collected by a questionnaire created by the authors in person during outpatient clinics as well as online. Questionnaire design was based on patient feedback and wider Craniofacial Team input. A mixture of short answer and multiple choice questions was used to collect data on each individual’s craniofacial condition and level of sporting activity. Data on parental anxiety was collected by self-reported scores on a 5-point Likert scale for various levels of contact sport (non-contact, light contact, heavy contact and combat sports). The 5-point Likert scale ranged from 1 (no anxiety) to 5 (very anxious). All data was anonymised. Data was collated by the authors using Google Forms and analysed using Microsoft Excel.

## Results

**Fig. 1:**
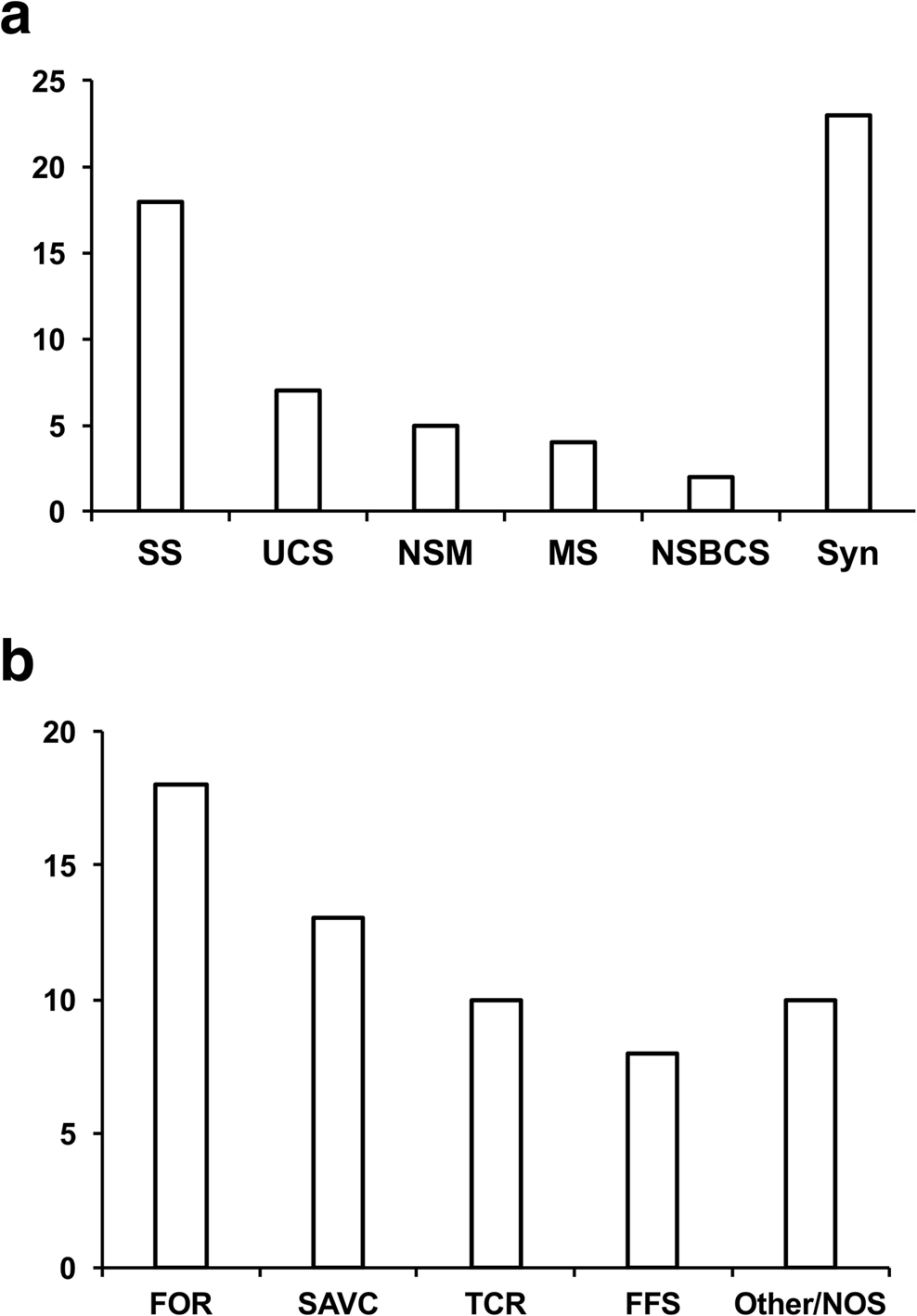
Diagnoses and operations for children of surveyed patients. **a** Craniofacial diagnoses (SS, sagittal synostosis; UCS, unicoronal synostosis; NSM, non-synostotic multisutural; MS, metopic synostosis; NSBCS, non-syndromic bicoronal synostosis; Syn, syndromic craniosynostosis). **b** Previous craniofacial operation, most recent if multiple surgeries (FOR, fronto-orbital remodelling; SAVC, spring-assisted vault cranioplasty; TCR, total calvarial remodelling; FFS, fronto-facial surgery including monobloc or bipartition)

One hundred forty-seven families were contacted (100 face to face, 47 via letter), of which 112 agreed to participate. Following exclusion of non-operated cases (i.e. new referrals being seen for first time in clinic, and those who had opted for conservative management, 30 families) and patients with non-craniosynostosis diagnoses (e.g. facial clefts, trauma, 23 families), there were 59 completed electronic surveys from parents of children with operated craniosynostosis. The mean age of participants at the time of survey was 7.8 years (range 3 months to 22 years). Thirty-six children had non-syndromic craniosynostosis (sagittal 18, unicoronal 7, non-syndromic multisutural 5, metopic 4, non-syndromic bicoronal 2), with 23 syndromic cases (Crouzon, Muenke, Apert, etc.; Fig. 1a). Prior surgical treatment included fronto-orbital remodelling (18), spring-assisted vault expansion (13) and others (Fig. 1b).

For the purposes of the survey, we divided sporting activity into intensity type and participation level. Intensity type was defined by the amount of contact: non-contact (e.g. gymnastics), light contact (e.g. soccer); heavy contact (e.g. rugby) and combat (e.g. boxing). Participation level was defined as follows: school mandatory, school optional (e.g. school clubs), non-school sports clubs, regional representative and national representative.

**Fig. 2:**
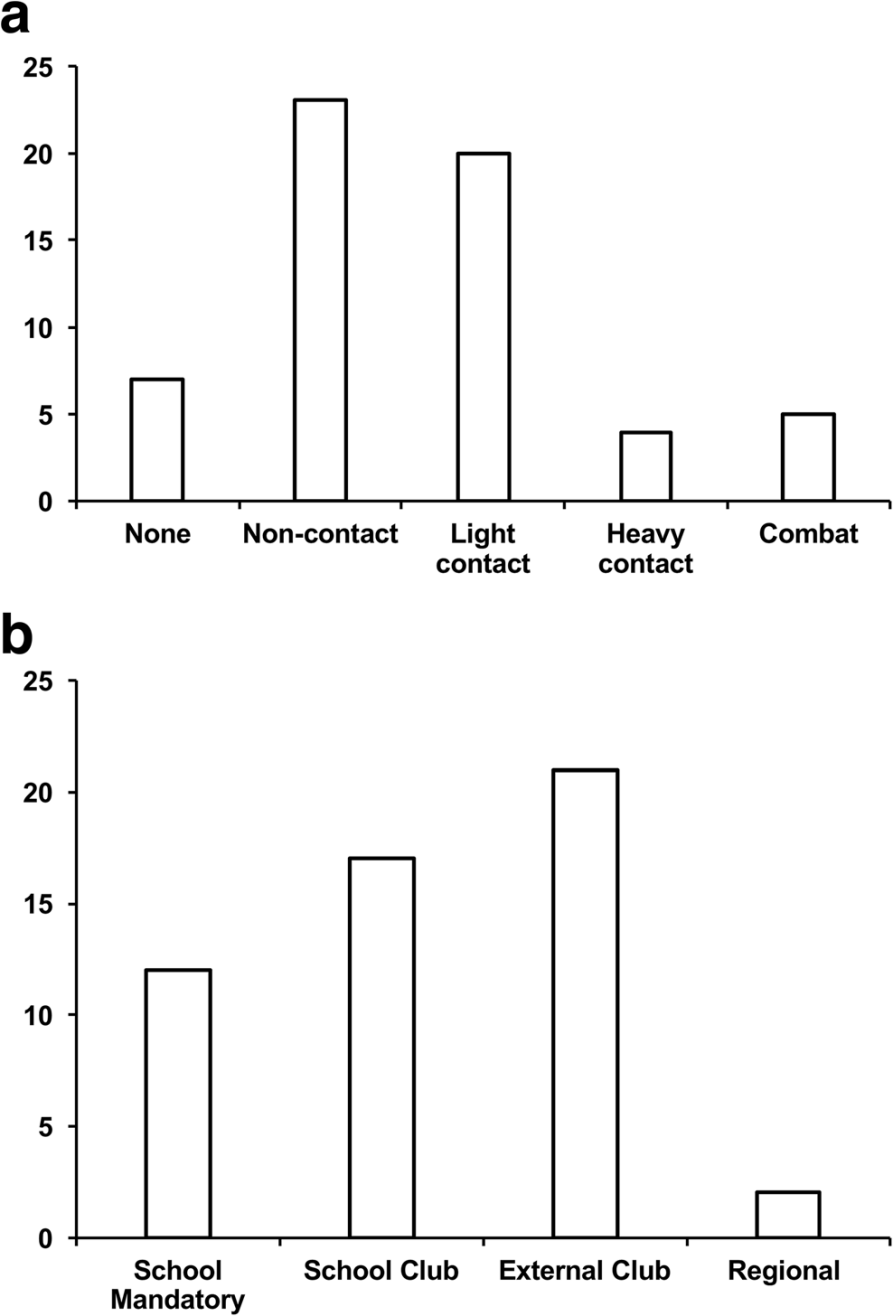
Intensity type and level of participation. **a** Most intense athletic activity undertaken: non-contact (e.g. gymnastics), light contact (e.g. soccer), heavy contact (e.g. rugby) and combat (e.g. boxing). **b** Highest level of participation. No children were competing at a national level

Of the 59 children surveyed, 52 were involved in sporting activity. Of these, the most intense type of sport undertaken was non-contact in 23, light contact in 20, heavy contact in 4 and combat in 5 (Fig. 2a). In terms of participation level, 12 were involved at school mandatory (physical education) only, 17 with school optional (school clubs), 21 with non-school sports clubs and 2 at regional representative level. No children were competing at national level (Fig. 2b). Only one child had been advised to avoid all sports (advice not from our Craniofacial Team).

**Fig. 3:**
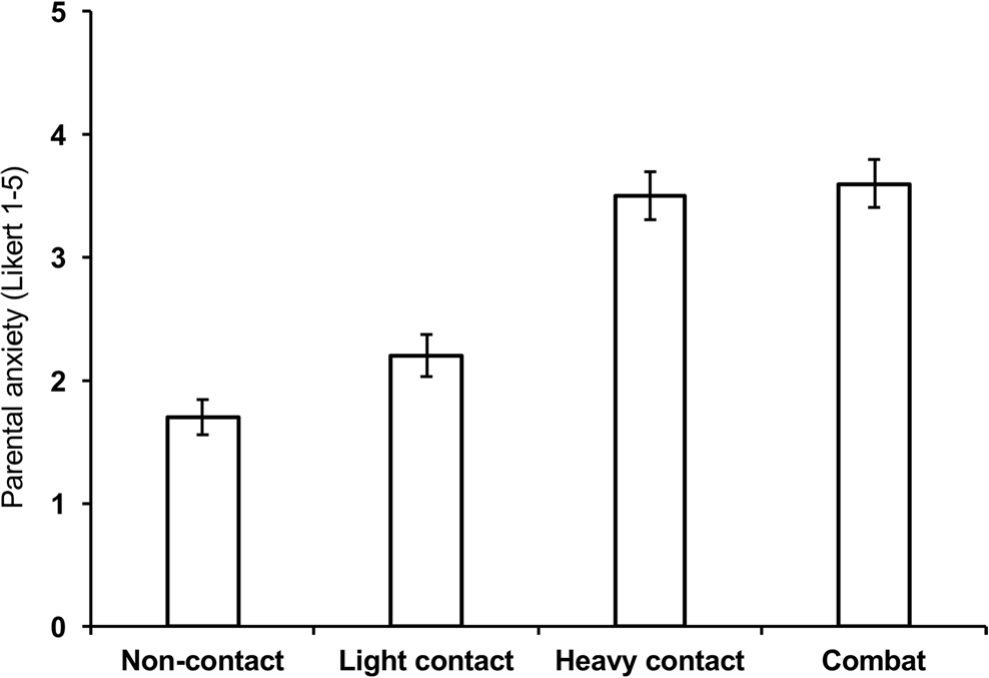
Parental anxiety increases with the prospect of more physically intense sporting activities. Five-point Likert scale, with 1 being no anxiety and 5 being extreme anxiety. Error bars = standard error of the mean (SEM)

Parental anxiety increased with increasing intensity type. Parents were asked about their level of anxiety of the prospect of their child undertaking the various intensity types, regardless of whether their child was currently undertaking that sport. Mean anxiety score (1–5 on a Likert scale) was 1.7 (standard error of the mean, SEM 0.14) for non-contact, 2.2 (SEM 0.17) for light contact, 3.5 (SEM 0.20) for heavy contact and 3.6 (SEM 0.20) for combat. Thirty-seven of 57 parents reported no anxiety (Likert 1) for non-contact sport, with only 1 having high (Likert 5) anxiety, increasing to only 1 no anxiety and 23 high anxiety for combat (Fig. 3). Subgroup analysis did not demonstrate any relationship between diagnosis (syndromic vs non-syndromic) or type of operation and level of anxiety.

Twenty-nine of 59 parents reported that they had been advice regarding sporting activity by the Craniofacial Team, of which 28 found the information useful. The 1 parent who did not find the information useful felt it was too generic and would have preferred more detailed advice. Of the 30 patients who did not report receiving advice, 16 stated that they would have appreciated being given advice. Fifteen parents reported that they had not allowed their child to undertake sports for a period of time after surgery, all of them quoting a concern about the potential for injury.

Four of 59 parents reported their child having sustained a sports-related injury: 3 head injuries and 1 ankle injury. None of the head injuries required hospital admission, and there were no lasting sequelae.

## Discussion

Anecdotally, we had noticed within the Craniofacial Team that parents of school-age children, who had undergone surgical craniosynostosis repair in infancy, were apprehensive about allowing their children to partake in sports—particularly when there was a perceived risk of head injury. However, an extensive search of the literature did not yield any publications addressing sports or physical activity after craniosynostosis surgery. A large cohort study had examined the incidence of traumatic brain injury in the 2 years after craniosynostosis surgery and found it to be 0 [9], but no other data is available.

This study indicates that the vast majority of children (88%) were taking part in some form of sporting activity and it was gratifying to find that only one was abstaining from sport on medical advice—but that advice was not from our Craniofacial Team but rather a physician from an external institution. In fact, 2 children were competing at regional representative level, indicating that previous craniosynostosis surgery is no barrier to athletic excellence.

Perhaps understandably, parental anxiety regarding sporting type increased as the contact sustained during play increased—presumably due to concerns about head injury. Despite these concerns, a number of children were partaking in heavy contact and combat sports, with little evidence of harm. Only 3 head injuries had been sustained in the series, and none of them had required hospitalisation or had any lasting effects.

Only half of parents had received specific advice about sports after craniosynostosis surgery; when it was given, this advice was almost unanimously valued. Since this study, we are making further efforts to discuss this important aspect with families in the craniofacial clinic and are developing written information to supplement this.

Limitations of the current study include its relatively small size and the potential for participation bias—as completing the questionnaire was optional, families with particularly high (or indeed low) levels of anxiety may have preferentially chosen to take part. Finally, we used a simple Likert scale of anxiety, rather than a validated tool or a face-to-face assessment with a psychologist. This was a practical decision based on the fact that it was unfeasible to carry out complex assessments on a large number of patients. However, for future studies it may be interesting to take a smaller group of families and examine their anxieties around sports in more detail.

Enjoyment of sport and physical activity is important for children’s physical and mental health, as well as the development of motor skills and the development of social bonds [3]. This study has illustrated that parents have anxiety regarding the prospect of their operated children with craniosynostosis undertaking athletic activity and that this anxiety increases with higher levels of physical contact. Despite this anxiety, the majority of appropriately aged children are taking part in sporting activity at various levels, and significant injury is not seen. Finally, parents appreciate being given advice about their children taking part in sports.

## Data Availability

Full data can be supplied on request.
